# New York Letter

**Published:** 1893-12

**Authors:** Wm. L. Russell

**Affiliations:** New York


### *CORRESPONDENCE.*

### OUR NEW YORK LETTER.

New York, November 15, 1893.

### INFANTILE PARALYSIS.

At the last regular monthly meeting of the Academy, Dr. A. B. Judson read a paper in reference to the prevention and cure of deformities from this cause, by means of orthopedic appliances.

In the discussion, Dr. Koplik said that the difficulty lay in telling what cases would recover and what require measures to prevent deformity. Dr. E. D. Fisher said that in his experience, treatment did not much affect the cord. In very young children, say twelve or eighteen months old, too early instrumental treatment was hardly advisable. He was accustomed then to wait for several months, in the meantime promoting nutrition by baths, massage and electricity. When the inflammatory stage had passed, and it became clear that locomotion was interfered with, or deformity was beginning, he advised instruments. Anything that helped the child to keep in motion was desirable, as motion, perhaps, helped the nutrition of the cord reflexly, and might be the means of saving some cells.

Dr. A. B. Townsend spoke of the difficulty of keeping the muscles in normal relation without the use of some apparatus for maintaining the limb in a straight position. If any muscles were paralyzed, the limb no longer remained straight, and deformity was sure to result.

Dr. A. D. Rockwell had seen most astonishing improvement result in some cases without the use of orthopedic appliances. He believed that the value of electricity in these cases had been overestimated; it had no influence in restoring the nerve elements, but it aided the nutrition of the limb, and perhaps affected the nerve cells reflexly. The muscles do not, as a rule, respond to faradic currants, and galvanism without interruption should be employed. When shortening and deformity had occurred appliances should be immediately resorted to.

Dr. H. L. Taylor believed that nearly all deformities resulting from these paralyses could be prevented. The problem was simply how to make the most of the mechanical possibilities of a set of damaged muscles. One thing was certainly gained by orthopedic appliances. Locomotion was made possible, and the child improved in all respects, both mentally and physically. After as long a period as fifteen years the muscular power had been found to remain good, and such limbs were well worth putting into a good position. He drew attention to the value of dry heat for improving the poor circulation. A hot box for the limb was employed by him, the temperature being kept 160° or 170° F., and the treatment applied for fifteen or twenty minutes, once or twice a day.

Dr. A. A. Phelps thought that no general practitioners ought to attempt to treat these cases after the child was two and a half years old, unless he was acquainted with mechanics. If the limb was placed in proper position, either with or without an operation, it could then be easily kept there by means of a light splint. In a case exhibited by him, he had divided the tendo Achillis, the planta fascia, and everything tense, and had procured useful feet, where a condition of equino-varus had existed, it being impossible to bring the heels down. The boy could now flex his foot well, and walk almost perfectly.

Dr. R. H. Sayre said that many cases could only be treated by the general practitioner, no specialist being available. It became the duty, then, of the practitioner, to familiarize himself sufficiently with the principles of mechanics to enable him to keep the bones in their proper relation. A little common sense would guide him sufficiently in most cases. It ought to be remembered that abnormal growth of bone resulted from abnormal pressure at certain points, with too little pressure at others. Thus bad knock-knee and club-foot, requiring severe operations for their correction, sometimes resulted. All such gross deformities could be avoided if the general practitioner but did his duty.

Dr. Jacobi said that he had hoped to hear something new in regard to the management of these cases, but had been disappointed. The use of the mechanical contrivances had been taught forty or fifty years ago. He thought it belonged to the orthopedists to instruct the general practitioner as to what specific measures he should pursue, rather than to simply urge upon him the necessity of turning over his cases to the specialist. His opinion was that in many cases it was sufficient to call the orthopedist in consultation occasionally. Very few cases recovered, and improvement took place very slowly. Contraction was seldom present before a month, and massage and electricity faithfully and patiently persisted with might accomplish a great deal. Passive motion and friction were also useful, and he had faith in strychnine used hypodermatically.

### MYXEDEMA.

The treatment of this disease by extracts of the thyroid glands of the lower animals has aroused almost as much interest as the discovery of Koch's tuberculin. It is to be hoped that expectations will be more fully realized this time. The results obtained thus far have certainly been remarkable, however inexplicable the action of the remedy may be.

A number of cases were reported at the Academy last evening, by Dr. G. W. Crary, who gave also a brief sketch of the history of the disease. In regard to its pathology he said that the thyroid gland was always found to be diseased, being atrophied and sclerosed. The skin was also the seat of changes, there being new connective tissue formation, and obliteration of glands, and desquamation of the superficial layers of cells. The arteries were atheromatous, and the heart hypertrophied, and affected with interstitial myocarditis. The nerves were the seat of a diffused neuritis. The red blood corpuscles were diminished, the white normal. The kidneys, liver and other glandular organs were found to present interstitial changes. The disease appeared at all ages, but was more common in very young infants, and in women at the menopause.

The clinical features of the disease were as follows: The face wa dull, heavy, mask-like, the skin being thickened and swollen at the eyelids and naso-labial folds. The nose was broad and flattened in appearance. The tongue was large and thickened, the fauces swollen and pale. A hectic flush was seen over the malar prominences, and the scaly skin was the seat of chloasma-like patches. The hands were swollen, the skin being thickened, bronzed or mahogany-like. The hair was coarse and thin, and sometimes absent in the axillæ, and over the pubes. The whole body was swollen, and the skin dry. The nails and teeth were imperfect. Pendulous fatty masses developed in various locations and the abdomen was large and pendulous. Dysphagia was a common symptom, and the patient snored much while sleeping. Breathing was slow, and dyspnea was not uncommon. The speech was monotonous and slow, and quite characteristic. The skin was cold, and subjective sensations of cold were frequent. Smell and taste were impaired. There was a paresis of the muscles, the movements being clumsy, and the walk waddling. Rheumatoid pains in the back and limbs frequently appeared. The temperature was subnormal, the pulse about seventy and weak. The specific gravity of the urine was low, urine being diminished in quantity, and albumen, and hyaline and granular casts being present. Mental depression was usually present. The disease pursued an essentially chronic course, death taking place usually from degeneration of the kidneys.

The condition described as cretinisn is probably infantile myxedema. It seemed to be endemic in certain localities in Europe. The condition might be present at birth, and if not it appeared before the age of five. The victims were dwarfed, seldom reaching more than three and a half feet, and from their unshapen heads and swollen features and forms, and clumsy gait were hideous to behold. The etiology of myxedema was obscure. It was often preceded by a drain on the organism, such as severe hemorrhage, excessive vomiting, frequent pregnancies, etc. A tubercular family tendency was sometimes noted.

The diagnosis was not usually difficult, though the general edematous appearance, and the presence of albumen and casts in the urine might lead to a diagnosis of chronic Bright's disease. The dysphagia however, the voice changes, and the peculiar facial appearance, with the elevated eyebrows, prominent naso-labial folds, and scanty hair were distinctive features.

The treatment consisted in the use of extracts of the thyroid glands of sheep. The credit of the introduction of this measure was due to Murray, of Newcastle-on-Tyne. The bad effects which had followed its early use, were due to the employment of too large doses, which produced flushing of the face, headache, nausea, with rise of temperature and acceleration of the pulse. Epileptiform convulsions had also been noted.

The effects of the remedy were very marked. The weight became reduced in the first two weeks, and the general appearance of the patient soon showed improvement. The urine increased in amount, the proportion of urea becoming greater, and exceeding that which could be accounted for the nitrogen in the food. The rheumatoid pains were often aggravated, and the anemia did not improve. It was well to keep the patient free from excitement, and to avoid depressing remedies. Rest in bed was desirable. In infants, the growth of the skeleton was accelerated by the treatment, and the mental capacity increased. After the limit of improvement had been reached small doses were required to keep the patient in health. The extract was prepared by adding twenty-four grains of the minced gland to a drachm of glycerine, and the dose was five to fifteen drops three times daily for adults, and one drop for infants. He had found the juice of the glands of lambs to be more active than those of older sheep.

Dr. M. A. Starr said that forty-six per cent. of the cases presented at the clinical society of London had exhibited mental disorder. Three of the seven seen by him had been brought because of these disorders. They had melancholia and were demented; there were no fixed delusions, however, and there was rather an apathetic condition with mental disability, than the usual forms of melancholia or dementia. He thought that there were possibly cases in hospitals for the insane, which could be cured by the treatment under consideration. Three of his cases had been under his observation for three years before the diagnosis was made; they were regarded by him, and by eminent consultants as cases of chronic Bright's disease. Dr. Starr exhibited a number of photographs showing the striking effects produced by the treatment, and the points of difference from Bright's disease.

Dr. W. G. Thompson spoke of the lack of knowledge concerning the functions of the thyroid gland, and of the value of the discovery of the treatment of myxedema in relation to this matter.

Dr. Kimball gave the history of a case treated by him with good results, by Parke, Davis & Co.'s capsules of thyroid extract.

In closing the discussion, Dr. Crary said that the temperature should not rise above 99° F. during the treatment. He thought careful study of all the symptoms necessary to distinguish some cases from Bright's disease, as extensive kidney lesions were frequently present.

WM. L. RUSSELL.

---

### TREATMENT OF INFANTILE CONVULSIONS.

M. Jules Simon recommends the following line of treatment of infantile convulsions: 1. Empty the digestive tract by an enema and by tickling the fauces to promote vomiting. 2. If the attack continues, administer ether or chloroform on a handkerchief. 3. Administer by the mouth, or if necessary by enemata, repeated doses of the following mixture: Chloral hydrate, fifteen grains; bromide of potassium, fifteen grains; syrup of codeia, ten drops; tincture of musk, ten drops; tincture of aconite, ten drops; orange-flower, three ounces and a half—this quantity to suffice for twenty-four hours. 4. When the attack is very grave give a warm bath and apply a small blister to the back of the neck or the epigastrium, leaving it on for three hours. Antiseptic precautions should be observed and a poultice subsequently applied.—*Canada Lancet.*

